# Kanakugiol, a Compound Isolated from *Lindera erythrocarpa*, Promotes Cell Death by Inducing Mitotic Catastrophe after Cell Cycle Arrest

**DOI:** 10.4014/jmb.1909.09059

**Published:** 2019-12-15

**Authors:** Jintak Lee, Hyun-Woo Chun, Thu-Huyen Pham, Jae-Hwan Yoon, Jiyon Lee, Myoung-Kwon Choi, Hyung-Won Ryu, Sei-Ryang Oh, Jaewook Oh, Do-Young Yoon

**Affiliations:** 1 Department of Bioscience and Biotechnology, Research Institute of Bioactive-Metabolome Network, Konkuk University, Seoul 05029, Republic of Korea; 2 Natural Medicine Research Center, Korea Research Institute of Bioscience and Biotechnology, Cheongju 8116, Republic of Korea; 3 Department of Stem Cell and Regenerative Biotechnology, Konkuk University, Seoul 05029, Republic of Korea

**Keywords:** Kanakugiol, breast cancer, necrosis, mitotic catastrophe, cell cycle arrest

## Abstract

A novel compound named ‘kanakugiol’ was recently isolated from *Lindera erythrocarpa* and showed free radical-scavenging and antifungal activities. However, the details of the anticancer effect of kanakugiol on breast cancer cells remain unclear. We investigated the effect of kanakugiol on the growth of MCF-7 human breast cancer cells. Kanakugiol affected cell cycle progression, and decreased cell viability in MCF-7 cells in a dose-dependent manner. It also enhanced PARP cleavage (50 kDa), whereas DNA laddering was not induced. FACS analysis with annexin V-FITC/PI staining showed necrosis induction in kanakugiol-treated cells. Caspase-9 cleavage was also induced. Expression of death receptors was not altered. However, Bcl-2 expression was suppressed, and mitochondrial membrane potential collapsed, indicating limited apoptosis induction by kanakugiol. Immunofluorescence analysis using α- tubulin staining revealed mitotic exit without cytokinesis (4N cells with two nuclei) due to kanakugiol treatment, suggesting that mitotic catastrophe may have been induced via microtubule destabilization. Furthermore, cell cycle analysis results also indicated mitotic catastrophe after cell cycle arrest in MCF-7 cells due to kanakugiol treatment. These findings suggest that kanakugiol inhibits cell proliferation and promotes cell death by inducing mitotic catastrophe after cell cycle arrest. Thus, kanakugiol shows potential for use as a drug in the treatment of human breast cancer.

## Introduction

Cancer is one of the most important threats to human health [1]. All tumor cells share a common characteristic, that of defective or failed mitosis leading to the generation of aneuploid or tetraploid cells [2, 3]. Theodor Boveri postulated more than 100 years ago that abnormal chromosome segregation during mitosis might induce tumor formation [4]. Breast cancer is among the most common causes of cancer-related deaths [5], and the worldwide incidence of breast cancer is highest among women [6]. Although significant progress has recently been achieved in early detection and diagnosis of breast cancer, the incidence of this cancer type remains high [7]. Breast cancers are generally resistant to therapeutics targeting apoptosis due to the status of tumor-suppressing gene products [8]. Therefore, therapies inducing other types of cell death are preferentially used in breast cancer treatment [9].

Various possibilities must be considered during the development of anticancer drugs. For instance, even though apoptosis induction is the most common target of anticancer drugs, some cancers still escape apoptosis by regulating apoptotic signals [10]. Recent advances in our understanding of cancer biology have enabled us to develop new targeting strategies in anticancer drug development to suppress tumor growth. Microtubule-stabilizing and -destabilizing agents are the two types of microtubule-targeting antimitotic drugs. Destabilizing agents, such as vinca alkaloids and colchicines, bind to one of the two tubulin classes, and thus prevent microtubule polymerization. However, most stabilizing agents bind to the taxoid binding site of β-tubulin, and result in enhanced microtubule polymerization [11].

Microtubules are important components of the cytoskeleton that are composed of α- and β-tubulin subunits, which dimerize to form linear protofilaments. These protofilaments then form the final microtubule structure. The dynamic nature of the microtubule plus ends with continuous polymerization and depolymerization allows them to form the cell structure, while enabling motility and intracellular transport [12]. Therefore, regulation of the microtubule function might be an effective alternative to inducing tumor cell death.

Uncontrolled cell proliferation and apoptosis evasion are typical tumor cell characteristics. Apoptosis is a mechanism of genetically programmed cell death, and is morphologically different from other cell death processes, which can be induced by various physiological and pathological stimuli [13]. Necrosis is an accidental cell death mechanism that generally occurs without activation of any signal transduction process. Interestingly, necrosis was recently shown to also occur through a programmed signaling pathway [14, 15]. Shah *et al*. demonstrated the presence of the 50 kDa major fragment of PARP in necrotic cell death [16]. Thus, this major fragment of PARP (50 kDa) is a necrotic signature. In 2012, the International Nomenclature Committee on Cell Death defined mitotic catastrophe as an intrinsic tumor-suppressing mechanism that detects mitotic failure, and drives a cell to an irreversible anti-proliferative death [17]. Although it does not directly induce cell death, mitotic catastrophe progresses by triggering anti-proliferative processes including apoptosis and necrosis to inhibit the proliferation of a cell with defective mitosis. Apoptosis-and necrosis-related morphological and biochemical characteristics can be observed during mitotic catastrophe due to the adoption of a variety of anti-proliferative pathways [12, 18]. Therefore, investigation of pathways related to apoptosis, necrosis, and mitotic catastrophe is recommended for finding new therapeutic cancer targets.

Kanakugiol, a compound recently isolated from *Lindera erythrocarpa*, was previously shown to have free radical-scavenging and antifungal activities [19, 20]. However, the information regarding the effect of kanakugiol on breast cancer cells remains unclear. Thus, there is a rationale for testing the anti-breast cancer effects of kanakugiol. Here, we demonstrate that a small molecule, kanakugiol, promotes cell death in breast cancer by inducing mitotic catastrophe after cell cycle arrest; thus, kanakugiol can be considered as a potential drug for treating breast cancer.

## Materials and Methods

### Instruments and Reagents

1D (^1^H, ^13^C, and DEPT) and 2D (COSY, HMQC, and HMBC) NMR spectra were obtained using Bruker AM 400 spectrometers (Bruker, USA) with tetramethylsilane (TMS) as the internal standard. HRESIMS (high-resolution electrospray ionization mass spectrometry) analysis was conducted on an ultraperformance liquid chromatography-quadrupole time-of-flight mass spectrometer (UPLC-QTOF-MS, Waters, USA) in the positive-ion mode.

### Extraction and Isolation

*L. erythrocarpa* fruits were collected from Jeju Island, Korea during October 2013. Dried fruits (5.0 kg) were subjected to methanol extraction (15 L × 2) three times at room temperature, to obtain solid extract (770.0 g). Next, 500 g of the extract was fractionated using a silica gel column (10 × 90 cm, JEO prep 60, 40-63 μm, 2.3 kg), and eluted using hexane−EtOAc mixtures (20:1→15:1→10:1→8:1→6:1→4:1→2:1→1:1) to obtain 10 pooled fractions (LE Frs. 1−10). These were then combined based on comparison of their TLC and UPLC-PDA profiles. LE Fr. 4 (105.5 g) with kanakugiol was isolated by column chromatography with reverse phase silica gel (11 × 90 cm, Zeoprep C18 75 μm, 4.0 kg), and eluted using MeOH/DW (40%→60%→80→100%) to isolate kanakugiol (4.3 g). Finally, purified kanakugiol was identified by comparing its MS and NMR spectral data with published literature [19].

### Spectroscopic and Physical Data for Kanakugiol

Yellow oil; UV (MeOH) λ_max_ nm 208, 314; ^1^H NMR (400 MHz, CDCl_3_) δ 7.91 (1H, d, *J* = 15.5 Hz, H-α), 7.82 (1H, d, *J* = 15.5 Hz, H-β), 7.62 (2H, m, H-2 and H-6), 7.40 (3H, m, H-3, H-4, H-5), 4.08 (3H, s, 2’-OCH_3_), 3.87 (6H, s, 3’-OCH_3_ and 5’-OCH_3_), 3.84 (3H, s, 4’-OCH_3_); ^13^C NMR (100 MHz, CDCl_3_) 194.0 (C- β’), 155.2 (C-2’), 153.8 (C-4’), 151.2 (C-6’), 144.4 (C-β), 138.7 (C-5’), 137.5 (C-3’), 135.4 (C-1), 130.7 (C-4), 129.2 (C-3 and C-5), 128.7 (C-2 and C-6), 126.7(C-α), 111.3 (C-1’), 62.4 (C-2’-OCH_3_), 61.8 (C-5’-OCH_3_), 61.6 (C-3’-OCH_3_), 61.3 (C-4’-OCH_3_); HRESIMS *m/z* [M+H]^+^ 345.1305,(calculated for C_19_H_21_O_6_, 345.1338).

### Cell Culture

The human breast cancer cell line MCF-7 was obtained from American Type Culture Collection (ATCC, USA). MCF-7 cells were cultured in DMEM medium (Welgene Inc., Korea) supplemented with heat-inactivated 10% (v/v) fetal bovine serum (FBS; Hyclone Laboratories, USA), and were cultured at 37°C in a humidified incubator containing 5% CO_2_.

### Assessment of Cell Morphology

Cells were seeded in a 6-well plate at a density of 3.0 × 10^5^ cells/well and incubated at 37°C overnight. After 24 h of incubation, various concentrations of kanakugiol were added to the cells, and they were incubated further for 24 h. Cell morphology was determined using an inverted phase-contrast microscope (Primovert; Zeiss, Germany).

### Cell Viability Assays

Cell viability was estimated using the 3-(4,5-dimethylthiazol-2-yl)-5-(3-carboxy methoxy phenyl)-2-(4-sulfophenyl)-2H-tetrazolium (MTS) assay. The MTS assay is a colorimetric assay used for quantifying the activity of mitochondrial reductase, which reduces the tetrazolium compound to formazan. MCF-7 cells (3×10^4^ cells/well) were seeded in 96-well plates containing 100 μl of the medium. They were then treated with various concentrations of kanakugiol. The effect of kanakugiol on cell viability was estimated using the CellTiter 96 AQueous One Solution Assay (Promega, USA), containing MTS and phenazine methosulfate, an electron-coupling reagent. An aliquot (20 μl) of the aqueous solution of the One reagent was added to each well, followed by incubation for another 1 h. The absorbance of the samples was measured at 492 nm using a microplate reader (Apollo LB 9110, Berthold Technologies GmbH, Germany). The percentage of viable cells was normalized relative to the untreated controls, and represented as the cell viability $(\%)=\times \frac{\left(\mathrm{OD}\mathrm{of}\mathrm{treated}\right)}{\left(\mathrm{OD}\mathrm{of}\mathrm{control}\right)}\times 100$ The viability assay was repeated three times.

### Annexin–Propidium Iodide (PI) Staining

Cells (3 × 10^5^ cells/well) were seeded into a 6-well plate and incubated for 24 h. Then, the cells were treated with kanakugiol in a dose-dependent manner and incubated at 37°C for a further 24 h. The cells were then washed and harvested using trypsin-EDTA. Finally, the cells were collected, and stained using Annexin V–fluorescein isothiocynate (FITC) Apoptosis Detection Kit (BD Bioscience, USA) for 15 min. The proportion of apoptotic cells was determined by using a Nov°Cyte Flow Cytometer (ACEA Biosciences, USA), and performing analysis using NovoExpress software (ACEA Biosciences).

### DNA Laddering

MCF-7 cells (9 × 10^5^ cells/well) were treated with doxorubicin or kanakugiol for 24 h, washed with ice-cold phosphate-buffered saline (PBS, pH 7.4), and harvested using trypsin-EDTA. The gDNA products were obtained using a G-spin Genomic DNA Extraction Kit according to the manufacturer’s instructions (iNtRON Biotechnology, Korea). DNA was loaded on 1.5% agarose gel and detected by electrophoresis.

### Reverse Transcription-PCR

Cells were lysed in 1 ml of solution with a easy-BLUE Total RNA Extraction Kit (iNtRON Biotechnology), and RNA was isolated according to the manufacturer’s instructions. Oligo (dT)-primed RNA (5 μg) was reverse transcribed using M-MuLV reverse transcriptase (New England Biolabs, USA). Reverse transcription polymerase chain reaction (RT-PCR) was performed on a PCR Thermal Cycler Dice (Takara Bio Inc., Japan) by using the following primer sets: *DR3* F 5’- CAG ATG TTC TGG GTC CAG GT-3’ and *DR3* R 5’-GCT GTC CAA GGG TGA CAG AT-3’, *FADD* F 5’-CAC AGA CCA CCT GCT TCT GA-3’ and *FADD* R 5’-CTG GAC ACG GTT CCA ACT TT-3’, *Fas* F 5’- ATA AGC CCT GTC CTC CAG GT-3’, *Fas* R 5’- TGG AAG AAA AAT GGG CTT TG-3’, *TRAIL* F 5’-GGA ACC CAA GGT GGG TAG AT-3’, *TRAIL* R 5’-TCT CAC ACT GCA ACC TC-3’, and *GAPDH* F 5’-GAG TCA ACG GAT TTG GTC GT-3’, *GAPDH* R 5’-GAC AAG CTT CCC GTT CTC AG-3’.

### Western Blotting

Cells were harvested using trypsin-EDTA and lysed in RIPA buffer (iNtRon Biotechnology,) at 4°C for 2 h. Cell lysates were clarified by centrifugation at 17,010 ×*g* and 4°C for 30 min. Protein concentrations were estimated using the Bradford assay (Bio-Rad, USA) and a UV/VIS spectrophotometer (Biowave; Biochrom, UK). Equal amounts of cell lysates were resolved by performing sodium dodecyl sulphate-polyacrylamide gel electrophoresis on 10–15% gels. Protein bands obtained were transferred onto polyvinylidene difluoride membranes (Cat No: IPVH00010; Millipore, USA). The membranes were blocked with Tris-buffered saline containing Tween-20 [TBST; 2.7 M NaCl, 54 mM KCl, 1 M Tris-HCl (pH 7.4), and 0.1% Tween-20], and 5% non-fat dried milk for 1 h at room temperature. Next, the membranes were incubated overnight at 4°C with primary antibodies (diluted 1:1000) specific to each target protein. After washing three times with TBST, the membranes were incubated with horseradish peroxidase-conjugated anti-rabbit or anti-mouse IgG secondary antibodies for 1 h at room temperature. After washing three times with TBST, the blots were detected using a WesternBright ECL Western Blotting Detection Kit (Advansta, USA). The following primary human monoclonal antibodies were used: poly (ADP-ribose) polymerase (PARP)aspase-9aspase-3axidcl-2cl-xL 2762s (all purchased from Cell Signaling Technology, USA), glyceraldehyde-3-phosphate dehydrogenase (*GAPDH*) sc-25778 (purchased from Santa Cruz Biotechnology, USA).

### Analysis of Mitochondrial Membrane Potential (MMP)

MMP (Δψm) was evaluated via JC-1 staining and flow cytometry. MCF-7 cells (9 × 10^5^ cells/well) were seeded into 3 ml medium in a 60 mm culture dish and treated with various concentrations of kanakugiol. The cells were harvested using trypsin-EDTA and transferred into 1.5 ml tubes. JC-1 (5 μg/ml) was added to the tube, and mixed until it was completely dissolved. Subsequently, the cells were incubated in the dark for 10 min at 37°C, centrifuged (300 ×*g*, 5 min, 4°C), washed twice with PBS, and resuspended in 200 μl PBS. The solutions were protected from light and analyzed by using Nov°Cyte Flow Cytometer (ACEA Biosciences, USA) and NovoExpress software (ACEA Biosciences).

### Cell Cycle Analysis

Approximately, 3 × 10^5^ cells/well were seeded into 6-well plates and incubated overnight. The cells were treated with different concentrations of kanakugiol and incubated further for 24 h. They were then washed, harvested, and fixed with ice-cold 70% ethanol at 20°C. After fixation, the cells were washed with PBS, and then stained with PBS containing 50 μg/ml PI (Propidium iodide) and 100 μg/ml RNase A for 30 min. The proportion of apoptotic cells was determined by performing flow cytometry analysis using a FACSCalibur device (BD Biosciences, USA), and CellQuest software (BD Biosciences).

### Immunofluorescence Staining

MCF-7 cells (3 × 10^5^ cells/well) were seeded into a 6-well plate, and incubated at 37°C overnight. The cells were then washed twice with PBS and treated with kanakugiol for 24 h. After treatment, the cells were fixed and permeabilized using 4%formaldehyde for 15 min at room temperature. Nonspecific sites were blocked by treatment with 1% bovine serum albumin (BSA) in PBS for 1 h at room temperature. Then, the cells were incubated overnight at 4°C with a mouse polyclonal primary antibody against α-tubulin (Santa Cruz Biotechnology) diluted 1:50 in PBS containing 0.1% BSA and washed three times with PBS (5 min/wash). Normal mouse IgG antibody was used as control. Next, the cells were incubated with FITC-labelled goat anti-mouse IgG secondary antibody (Merck Millipore, Germany) diluted 1:200 in PBS containing 0.1% BSA for 1 h at room temperature. After washing twice with PBS, the cells were stained with 4,6-diamidino-2-phenylindole (DAPI; Sigma-Aldrich, USA) for 10 s at room temperature. Fluorescence images were obtained using an BX61-32FDIC upright fluorescence microscope (Olympus, Japan) equipped with the 100× objective lens.

### Statistical Analyses

One-way analysis of variance (ANOVA) was conducted. Three independent experiments were performed. The data are expressed as mean ± standard deviation (SD, *n* = 3). A *p*-value < 0.05 was considered statistically significant.

## Results and Discussion

### Kanakugiol Reduced Breast Cancer Cell Viability

**Fig. 1:**
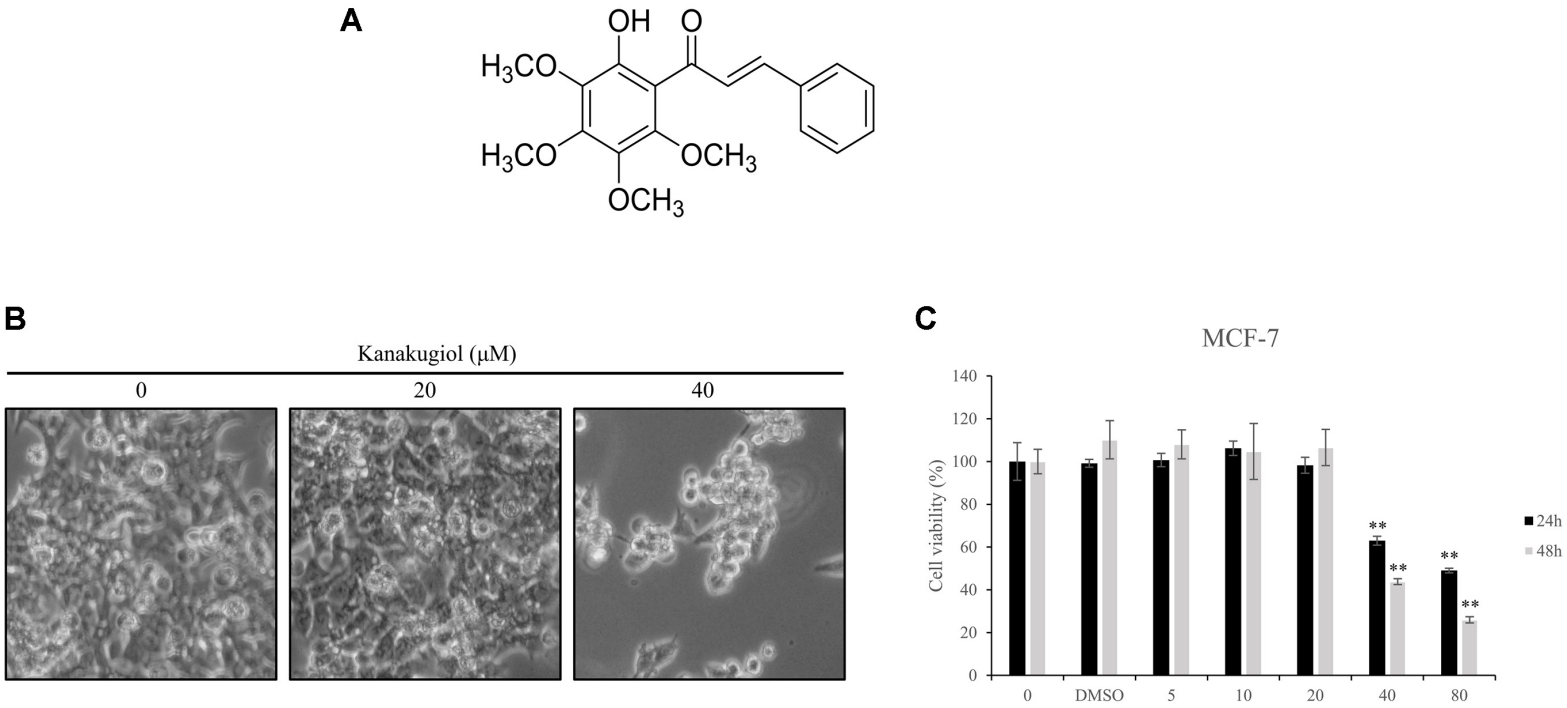
The inhibitory effect of kanakugiol on the viability of MCF-7 breast cancer cells. (**A**) Chemical structure of kanakugiol. (**B**) Micrographs of MCF-7 cells treated with the indicated concentrations of kanakugiol. (**C**) MCF-7 cells were treated with the indicated concentrations of kanakugiol for 24 h and cell viability was assessed by the MTS assay. Data are presented as mean ± standard deviation (*n* = 3). **p < 0.005 versus control cells.

Free radical-scavenging and antifungal activities of kanakugiol have been previously reported [19, 20]. Based on these findings, we investigated the potential anti-cancer effects of kanakugiol (Fig. 1A). For this purpose, we quantified the effects of kanakugiol on the growth of human MCF-7 breast cancer cells. MCF-7 breast cancer cells were treated with various concentrations of kanakugiol for different time periods. Optical microscopy analyses showed a decrease in the proportion of viable MCF-7 cells upon kanakugiol treatment (Fig. 1B). We also determined the effect of kanakugiol on MCF-7 cell viability using the MTS assay. The viability of MCF-7 breast cancer cells decreased in a dose- and time-dependent manner upon kanakugiol treatment (Fig. 1C). Our data thus suggest that kanakugiol-mediated MCF-7 cell death.

### Kanakugiol Induced Cell Death by Mediating Necrosis with Partial Intrinsic Apoptotic Pathways

**Fig. 2:**
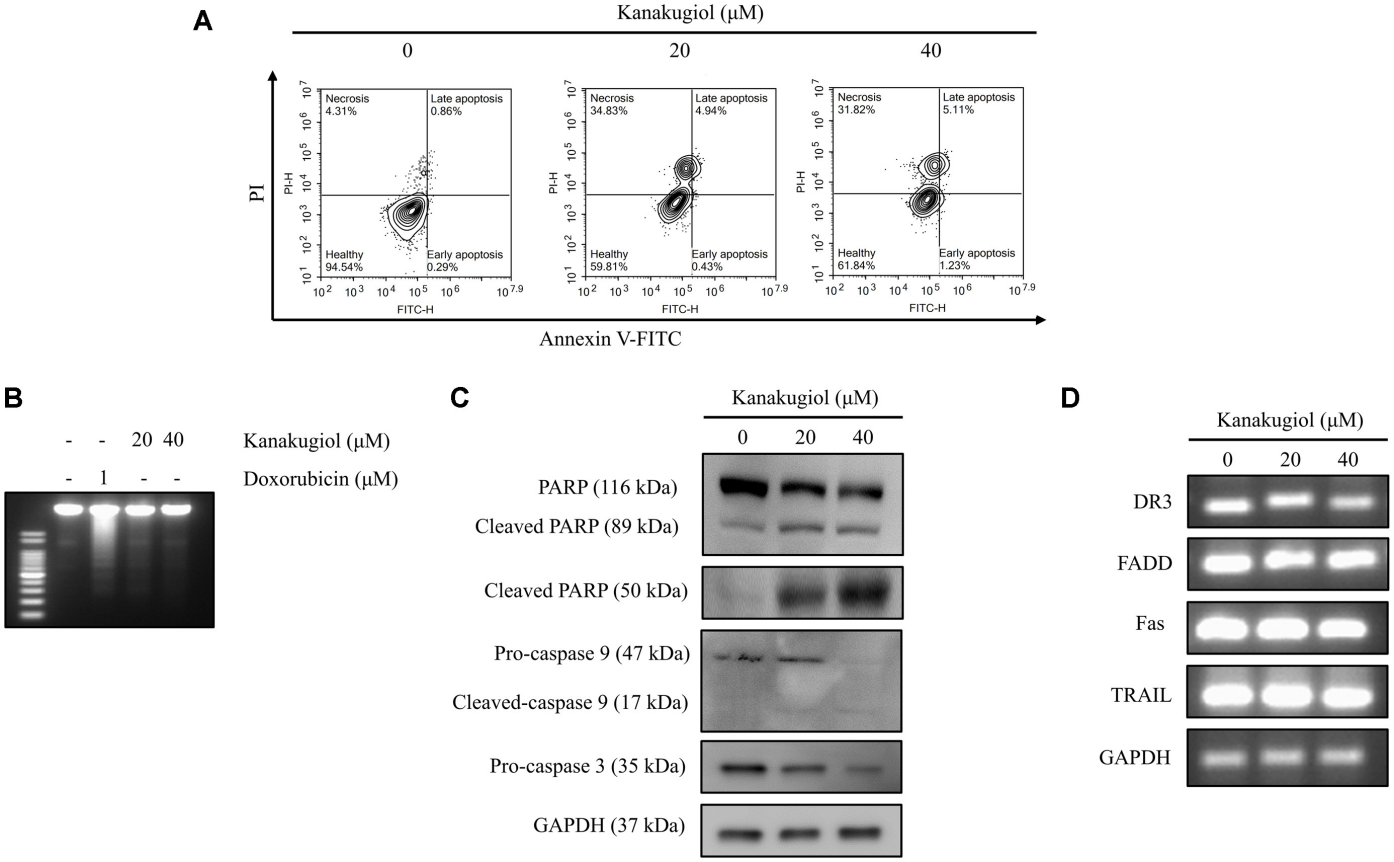
The effects of kanakugiol on necrosis and apoptosis in MCF-7 breast cancer cells. (**A**) MCF-7 cells were treated with varying doses of kanakugiol for 24 h. Apoptosis/necrosis was detected by a flow cytometer using Annexin-V/ PI staining as described in Methods section. (**B**) MCF-7 cells were treated with doxorubicin or kanakugiol for 24 h and the gDNA products were loaded onto 1.5% agarose gel. DNA fragmentation was not induced by kanakugiol-treated MCF-7 cells. (**C**) Western blot analyses to determine the expression levels of the intrinsic apoptotic markers. (**D**) Expression levels of death receptors as detected by PCR.

We next sought to clarify whether the kanakugiol-mediated cell death was due to apoptosis. Apoptosis is a cell death process that is important in maintaining the balance of cell death and proliferation [21]. Upon induction of apoptosis, phosphatidylserine, a component of the cell membrane, performs a flip-flop transition from the inner to the outer membrane [22]. Annexin V–PI staining assay revealed non-significant alterations in early (1.23%) and late apoptosis (5.11%) levels in kanakugiol-treated cells. However, necrosis levels increased dramatically (31.82%)(Fig. 2A).

Doxorubicin induces apoptosis by activating a variety of apoptotic signals [23]. DNA fragmentation is one of the most specific features of apoptosis. Here, a DNA laddering pattern is formed due to internucleosomal DNA cleavage [24]. Unlike kanakugiol, doxorubicin induced DNA laddering (Fig. 2B). Furthermore, kanakugiol treatment caused an increase in the levels of cleaved PARP (50 kDa), a major PARP fragment observed in necrotic cell death [16].

**Fig. 3:**
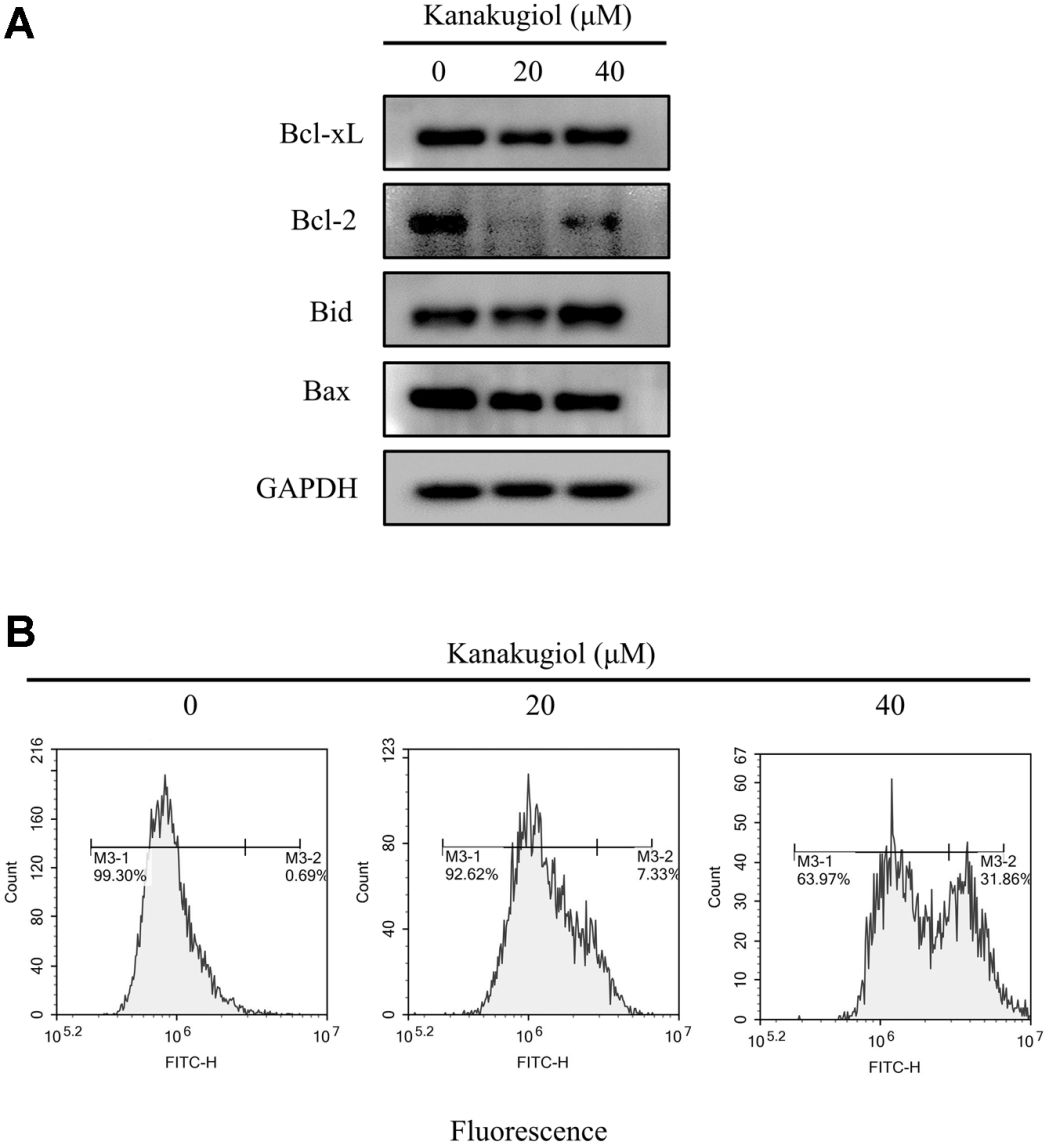
Effects of kanakugiol on factors involved in the function of mitochondria in MCF-7 breast cancer cells. (**A**) Western blots of mitochondrial Bcl-2 family in MCF-7 cells. (**B**) Kanakugiol induced mitochondrial membrane potential collapse. The difference in JC-1 colors was analyzed by flow cytometry.

The intrinsic and extrinsic pathways are the two types of apoptotic pathways. Mitochondrial functions are closely associated with the intrinsic pathway [25]. The family of Bcl-2 proteins is an important component of apoptotic pathway. The main function of Bcl-2 family members is to regulate apoptosis [26]. The Bcl-2 protein family includes two groups: anti-apoptotic Bcl-2, Bcl-xL and pro-apoptotic Bid, Bax, and Bad proteins [27]. We investigated the effect of kanakugiol on factors related to the mitochondrial intrinsic pathway. Bcl-2 expression was suppressed (Fig. 3A). The cleavage of apoptotic factors was slightly enhanced, and mitochondrial membrane potential collapsed in kanakugiol-treated MCF-7 cells (Figs. 2C and 3B). The levels of death receptor family factors, which are associated with the extrinsic apoptotic pathway [27], remained unaffected in kanakugiol-treated cells (Fig. 2D).

**Fig. 4:**
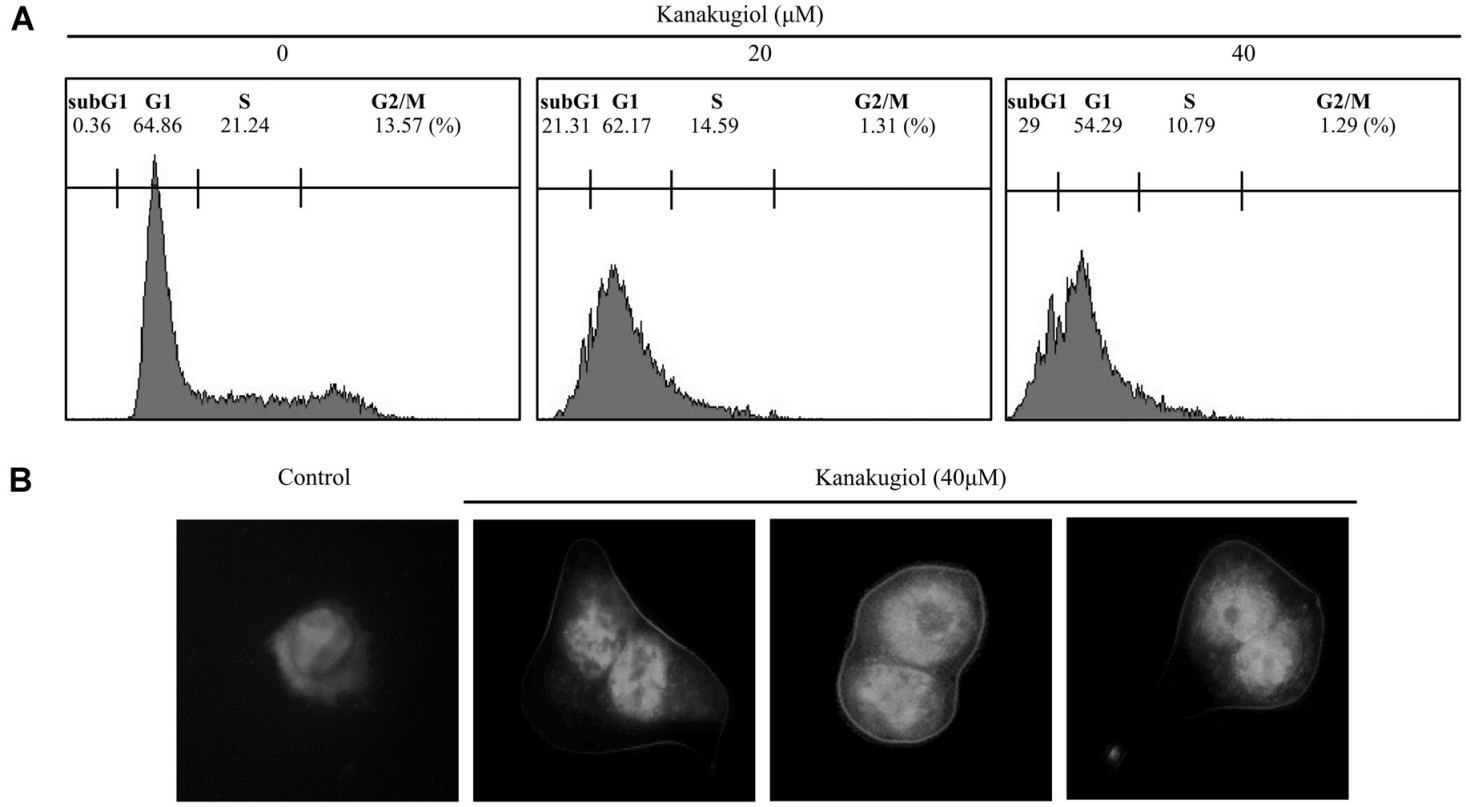
Effects of kanakugiol on cell cycle progression and mitotic catastrophe (MC) in MCF-7 breast cancer cells. (**A**) Cell cycle analysis by FACS using PI-staining. (**B**) Images of nuclear morphology and microtubule filaments were determined by performing DAPI and α-tubulin staining in kanakugiol-treated MCF-7 cells. Micrographs were taken using a fluorescence microscope (magnification, 100×).

Necroptosis, one mode of programmed necrosis, is a planned and genetically programmed type of cell death. RIPK1, RIPK3 (Receptor interacting kinase 1, 3) and MLKL (Mixed lineage kinase domain-like pseudokinase) are necroptosis factors that play significant roles in necroptosis pathway [28]. To investigate whether kanakugiol-induced necrosis type is generalized necrosis or necroptosis, we checked the mRNA expressions of the factors by RT-PCR. The data showed no increase in the levels of the factors (Fig. 4S); therefore, kanakugiol-induced necrosis type is generalized necrosis, not necroptosis. Taken together, cell death was induced via necrosis with partial mitochondrial intrinsic apoptotic pathways upon kanakugiol treatment in MCF-7 cells.

### Kanakugiol Induced Mitotic Catastrophe after Cell Cycle Arrest in MCF-7 Cells

**Fig. 5:**
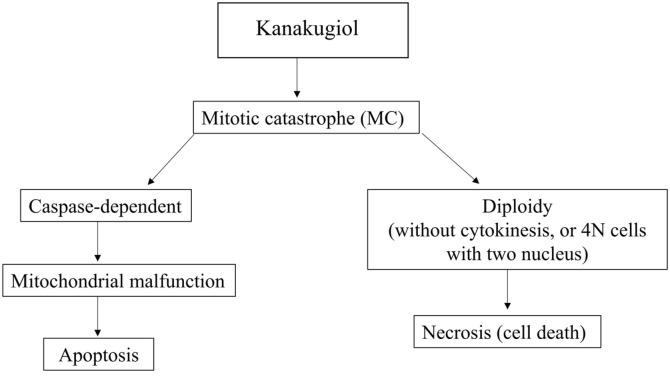
Mimetic diagram of kanakugiol efficacy pathway in MCF-7 cells. Kanakugiol inhibits proliferation of MCF-7 cells through mitotic catastrophe. Kanakugiol can induce major necrosis and minor apoptosis.

Cell death and proliferation are closely associated with cell cycle [29]. The specific cell cycle phase can be identified by flow cytometry which precisely quantifies the cellular DNA content. DNA fragmentation is one of the typical features of apoptotic cells, and PI-staining assay is commonly used for cell death detection [30]. Cell cycle analysis demonstrated that kanakugiol induced cell cycle arrest in sub-G1 phase in MCF-7 cells (Fig. 4A). The proportion of cells in sub-G_1_ phases increased upon kanakugiol treatment. Mitotic catastrophe denotes cell death due to delayed mitosis. In order to obtain more direct evidence regarding mitotic catastrophe, we performed an immunofluorescence assay for staining α-tubulin. Immuno-fluorescence results showed the abnormal structures of microtubules and diploidy in kanakugiol-treated cells (Fig. 4B). Taken together, kanakugiol induced mitotic exit without cytokinesis (4N cells with two nuclei), suggesting that mitotic catastrophe may have been induced via microtubule destabilization. Furthermore, mitotic catastrophe may also have been induced after cell cycle arrest in MCF-7 cells. These findings suggest that kanakugiol inhibits cell proliferation and promotes cell death by inducing mitotic catastrophe after cell cycle arrest. Thus, kanakugiol shows potential for use as a drug in human breast cancer treatment. A model summarizing the effects of kanakugiol is presented in Fig. 5.

## Supplemental Materials



Supplementary data for this paper are available on-line only at http://jmb.or.kr.
