# Supplementary material for: Kanakugiol, a Compound Isolated from Lindera erythrocarpa, Promotes Cell Death by Inducing Mitotic Catastrophe after Cell Cycle Arrest
Source: J Microbiol Biotechnol. 2019 Dec 15;30(2):279–86. doi: 10.4014/jmb.1909.09059 (PMC9728372; doi:10.4014/jmb.1909.09059)
Supplement: Supplementary file 1 [file JMB-30-2-279-supple.pdf]

J. Microbiol. Biotechnol.
https://doi.org/10.4014/jmb.1909.09059

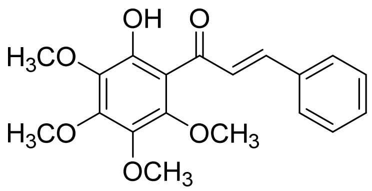

2. MATERIALS AND METHODS

2.1. Instruments and reagents

1D (^{1}H, ^{13}C, and DEPT) and 2D (COSY, HMQC, and HMBC) NMR spectra were obtained on Bruker AM 400 spectrometers (Bruker) with tetramethylsilane (TMS) as the internal standard. HRESIMS were measured on an ultraperformance liquid chromatography quadrupole time of flight mass spectrometer (UPLC-QTOF-MS, Waters, Milford, MA, USA) in the positive-ion mode.

2.2. Extraction and isolation

During October 2013, the fruits of *L. erythorocarpa* were collected from Jeju Island, Korea. The dried fruits of *L. erythorocarpa* (5.0 kg) a was extracted three times with methanol (15 L × 2) at room temperature to obtain 770.0 g of solid extract. The extracts (500.0 g) was fractionated on a silica gel column (10 × 90 cm, JEO prep 60, 40-63 μm, 2.3 kg) and eluted using hexane−EtOAc mixtures (20:1→15:1→10:1→8:1→6:1→4:1→2:1→1:1) to give 10 pooled fractions (LE Frs. 1−10), which were combined based on comparison of their TLC

and UPLC-PDA profiles. LE Fr. 4 (105.5 g) enriched with kanakugiol was isolated by column chromatography on revers phase lica gel (11 × 90 cm, Zeoprep C18 75 μm, 4.0 kg) and eluted using MeOH/DW (40%→60%→80→100%] to yield kanakugiol (4.3 g). Finally, the purified kanakugiol was identified by comparing its MS and NMR spectral data with published literature (Lee et al., 2015).

2.2.1. Kanakugiol

Yellow oil; UV (MeOH) λ_{max} nm 208, 314; ^{1}H NMR (400 MHz, $CDCl_3$) δ 7.91 (1H, d, J = 15.5 Hz, H-α), 7.82 (1H, d, J = 15.5 Hz, H-β), 7.62 (2H, m, H-2 and H-6), 7.40 (3H, m, H-3, H-4, H-5), 4.08 (3H, s, 2′-OCH_3), 3.87 (6H, s, 3′-OCH_3 and 5′-OCH_3), 3.84 (3H, s, 4′-OCH_3); ^{13}C NMR (100 MHz, $CDCl_3$) 194.0 (C- β′), 155.2 (C-2'), 153.8 (C-4′), 151.2 (C-6'), 144.4 (C-β), 138.7 (C-5′), 137.5 (C-3′), 135.4 (C-1), 130.7 (C-4), 129.2 (C-3 and C-5), 128.7 (C-2 and C-6), 126.7(C-α), 111.3 (C-1′), 62.4 (C-2′-OCH_3), 61.8 (C-5′-OCH_3), 61.6 (C-3′-OCH_3), 61.3(C-4′-OCH_3); HRESIMS m/z $[M+H]^+$ 345.1305, (calculated for $C_{19}H_{21}O_6$, 345.1338).

ACKNOWLEDGMENT

This work was supported by the KRIBB Research Initiative Program (KGM1221814).

REFERENCE

Identification of plant compounds that disrupt the insect juvenile hormone receptor complex. Proc Natl Acad Sci U S A. 10; 112(6): 1733-8.

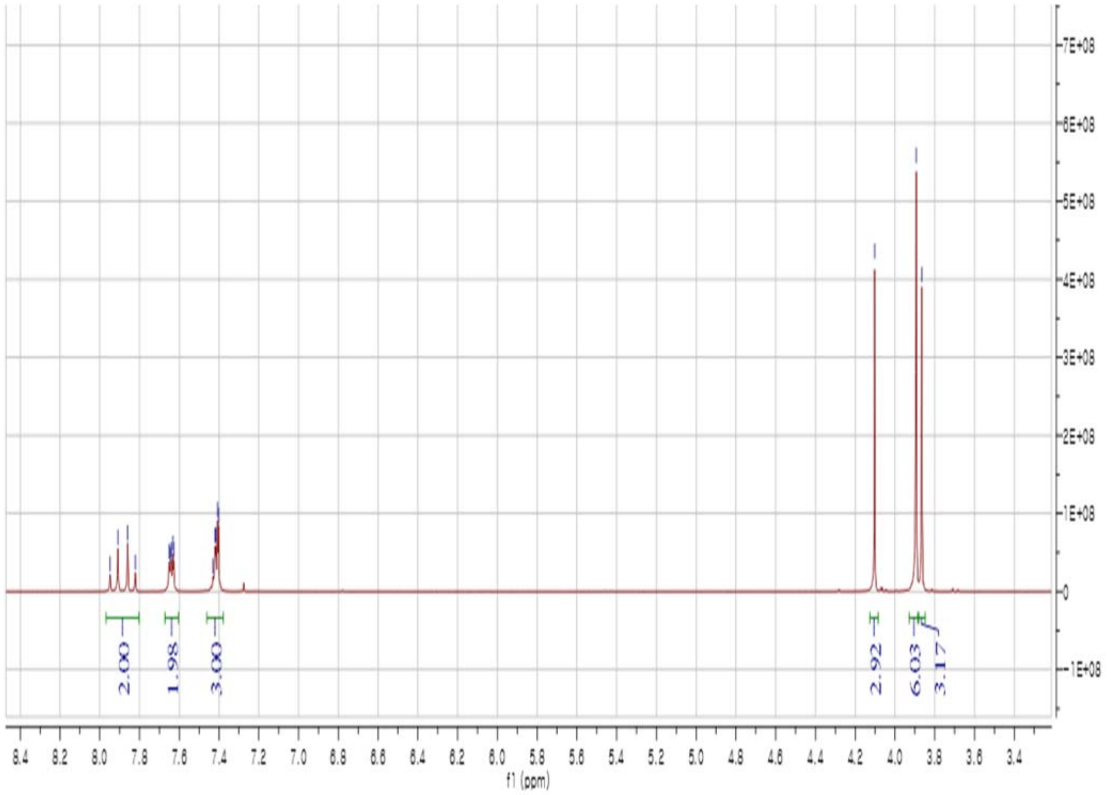

Figure 1S. ^{1}H-NMR (400 MHz, $CDCl_3$) spectrum of Kanakugiol.

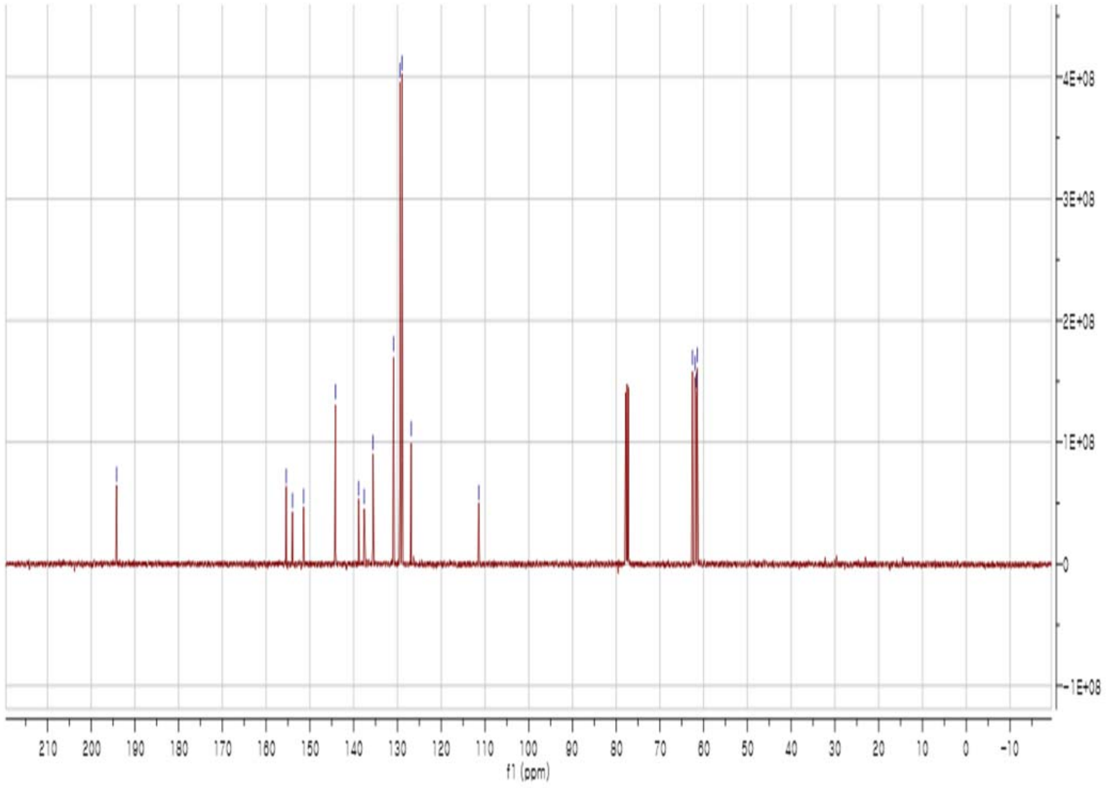

Figure 2S. ^{13}C-NMR (100 MHz, $CDCl_3$) spectrum of Kanakugiol.

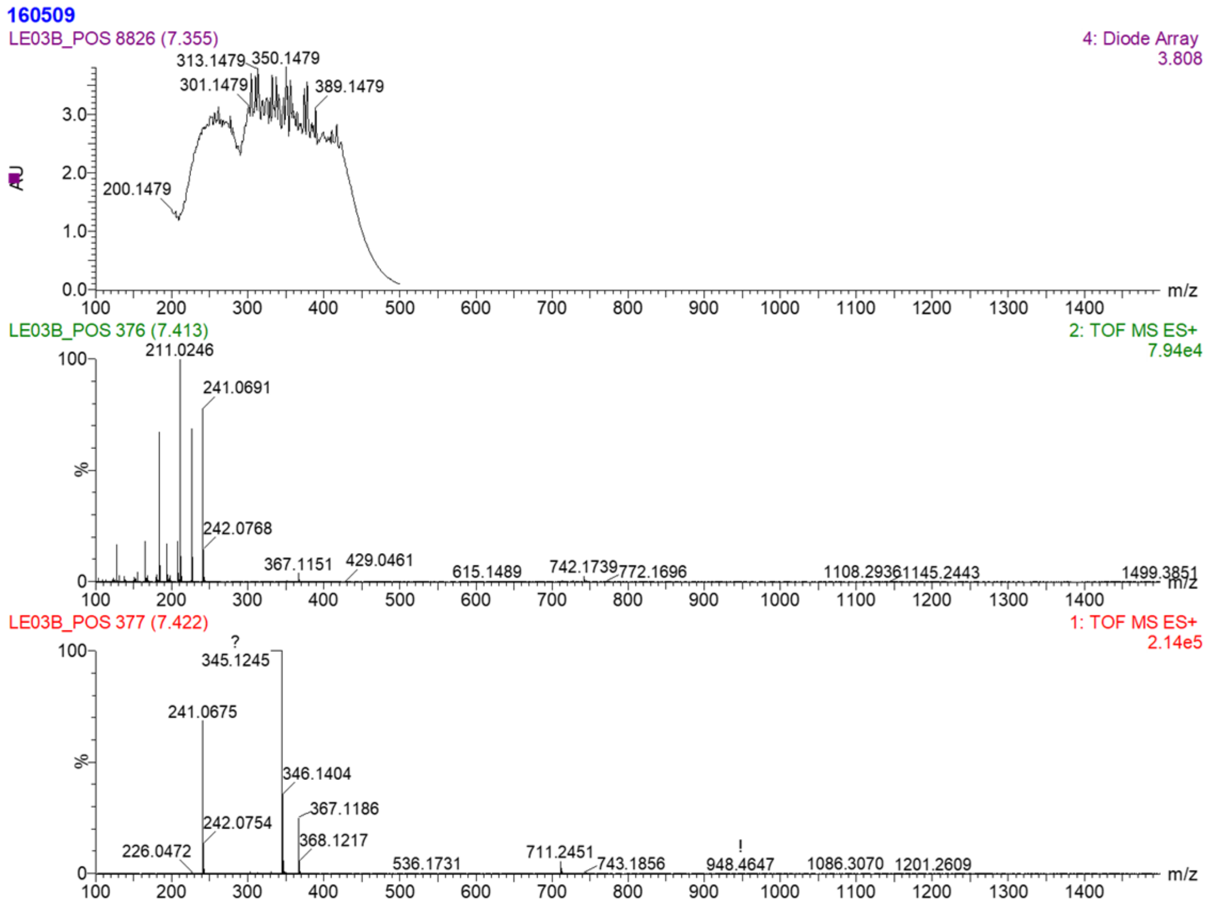

Single Mass Analysis

Tolerance = 10.0 PPM / DBE: min = -1.5, max = 50.0

Element prediction: Off

Number of isotope peaks used for i-FIT = 3

Monoisotopic Mass, Even Electron Ions

67 formula(e) evaluated with 2 results within limits (up to 50 best isotopic matches for each mass)

Elements Used:

Mass	Calc. Mass	mDa	PPM	DBE	Formula	i-FIT	i-FIT Norm	Fit Conf %	C	H	O
345.1305	345.1279	2.6	7.5	18.5	C26 H17 O	322.3	0.007	99.26	26	17	1
	345.1338	-3.3	-9.6	9.5	C19 H21 O6	327.2	4.907	0.74	19	21	6

Figure 3S. UPLC-QTof-MS and HREIMS data of Kanakugiol.

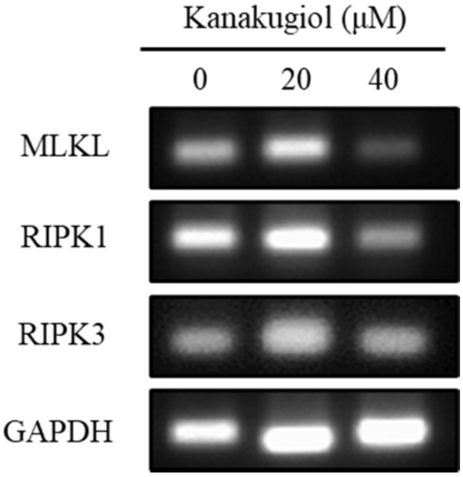

Figure 4S. The mRNA expressions of the necroptosis factors in MCF-7 breast cancer cells.
